# Axon hillock currents enable single-neuron-resolved 3D reconstruction using diamond nitrogen-vacancy magnetometry

**DOI:** 10.1038/s42005-020-00439-6

**Published:** 2020-10-02

**Authors:** Madhur Parashar, Kasturi Saha, Sharba Bandyopadhyay

**Affiliations:** 1 School of Medical Science and Technology, Indian Institute of Technology Kharagpur, Kharagpur, West Bengal 721302, India; 2 Department of Electrical Engineering, Indian Institute of Technology Bombay, Powai, Mumbai, Maharashtra 400076, India; 3 Department of Electronics and Electrical Communication Engineering and Advanced Technology Development Centre, Indian Institute of Technology Kharagpur, Kharagpur, West Bengal 721302, India

## Abstract

Sensing neuronal action potential associated magnetic fields (APMFs) is an emerging viable alternative of functional brain mapping. Measurement of APMFs of large axons of worms have been possible due to their size. In the mammalian brain, axon sizes, their numbers and routes, restricts using such functional imaging methods. With a segmented model of mammalian pyramidal neurons, we show that the APMF of intra-axonal currents in the axon hillock are two orders of magnitude larger than other neuronal locations. Expected 2D magnetic field maps of naturalistic spiking activity of a volume of neurons via widefield diamond-nitrogen-vacancy-center-magnetometry were simulated. A dictionary-based matching pursuit type algorithm applied to the data using the axon-hillock’s APMF signature allowed spatiotemporal reconstruction of action potentials in the volume of brain tissue at single cell resolution. Enhancement of APMF signals coupled with magnetometry advances thus can potentially replace current functional brain mapping techniques.

The development of in vivo two-photon calcium imaging^1^ and subsequent development of fast voltage/calcium sensors^2,3^ of neuronal membrane potential has allowed probing of local neuronal circuits^4–8^ in the mammalian brain at single-cell and millisecond spatiotemporal resolution. The advent of this technique actively triggered the study of excitatory–inhibitory populations of neurons and their functional connectivity^9–11^ in the passive sensory and active behavioral state of an organism. Multiphoton functional neuronal imaging is limited to depths of a maximum of 1mm from the brain surface due to limits of optical penetration of deep tissue and scattering^1,5,12,13^. Therefore, local neuronal populations from deep areas of the brain, like the hippocampus, many regions of the frontal cortex, amygdala can not be probed at the local circuitry level, unless largely invasive and potentially damaging optical fibers are used.

Methods for ultrasensitive microscale magnetic field sensing^14–21^ or single-cell resolution functional magnetic resonance imaging^22,23^ are being progressively developed to address this challenge. Diamond-nitrogen-vacancy centers (NVC) have emerged as a class of ultrasensitive nanoscale magnetic field detectors that function at ambient temperature^24–26^. Additionally, the NVC’s inert chemical nature allows it to be placed very close to the biological tissue^27^ allowing sensitive probing of biological magnetic fields^21^. In this context, Barry et al.^28^ experimentally demonstrated the measurement of worm axon action potential (AP)-associated magnetic field (APMF), which was found to be ~600 pT peak-to-peak in magnitude, using an ensemble of 2D NVC layer in the diamond. Notably, high-sensitivity microscale magnetic field mapping has been made possible by the preparation of high-density NVC diamond samples with high intrinsic coherence^14^. Further, the directional orientation of different NVC along the tetrahedral axes is used to obtain the vector direction of magnetic field^28,29^. APMF signal bandwidth falls in direct current (DC) to a few kilohertz bandwidths. DC-field sensitivity in NVC experiments is rapidly improving toward quantum projection noise limit^28^, at ~100 fTHz^$-\frac{1}{2}$^, with the best DC sensitivity record to be measurement at 50 pT Hz^$-\frac{1}{2}$^. for ensemble vector magnetometry measurements^30^. With constantly improving DC-field sensitivity, the community is expected to capture mammalian neuronal spike signals using diamond NVCs.

However, a challenge in probing a network of mammalian neurons in vitro or in vivo will be to reconstruct AP timing and location of single neurons from diamond NVC magnetometry. Previous work in the reconstruction of simulated APMF recordings via diamond NVC magnetometry has been restricted to simple models of passive conducting axons via filtering of noise in spatial frequency domain^28^ and a Wiener filter-based reconstruction of axonal firing^31^. Also, theoretical work has been carried out to develop inverse filters^32–34^ for the reconstruction of two cylindrical axonal currents, where analytical expression for current density was known. To the best of our knowledge, no method has been developed that takes into account the complex geometry and physiology of cortical neurons, where analytical expression for intra-axonal currents can not be derived. Further, single-AP-event detection from a time series of measurements must also be combined with spatial reconstruction, a necessary feature that is absent in previous studies.

In this work, we address the reconstruction of spike location and timing for realistic mammalian cortical pyramidal neurons, comprising of soma, axon-hillock region, axon initial segment, and other regions, specifically with respect to the case of measuring 2D vector magnetic field map (referred as diamond–nitrogen-vacancy magnetometric maps (NVMM) further in this text) via widefield diamond NVC magnetometry. We simulated voltage propagation in a realistic cortical pyramidal neuron model^35^ and obtained intra-axonal current profiles during an AP. These spatiotemporal current profiles were used to estimate the vector magnetic field during an AP. We found a 36 pT peak-to-peak mammalian APMF magnitude, which is close to the current limits of diamond NVC-based DC magnetometers. Notably, we found that axon hillock contributes almost two orders more, as compared to other axonal regions, to the measured APMF estimate. This naturally occurring advantage simplifies the inverse problem from being equivalent to solving randomly oriented current-carrying wires, where the location of ultra-small current keeps changing in 3D space as the AP propagates over hundreds of microns, to primarily a set of ~10-μm-sized axon-hillock region, fixed in space and exhibiting localized current flow only when an AP occurs in the corresponding cell soma. We propose an adaptation of dictionary-based matching pursuit algorithm^36–39^, to be applied on measurements from widefield diamond NVC magnetometry, for solving individual spike timings and locations in a 3D volume of randomly oriented pyramidal neurons. We show that the reconstruction of randomly oriented neurons in 3D can be achieved with accuracy ~70% and also for neurons arranged in a 2D plane parallel to the NVC layer. We find that our matching pursuit-based algorithm allows high noise resilience to the reconstruction. Further, we analyze the closest distance between a pair of cells that can be resolved by the algorithm. We find that nearby single neurons spiking near simultaneously with time difference 1 ms or more can be reliably resolved. Based on reconstruction errors, we infer that strongly correlated columns of the dictionary, due to the similarity of magnetic field patterns formed by two closely located neurons, are the main constraints to achieving perfect reconstruction.

## Results

### High magnetic field contribution of axon-hillock currents

APMF of worm (marine fanworm *Myxicola infundibulum* and the North Atlantic longfin inshore squid Loligo pealeii) single axons were estimated to be ~600 pT in magnitude measured with ensemble diamond NVC imaging setup^28^. However, mammalian neurons have a significantly smaller cross-sectional diameter (~1 μm at nodes) and carry orders of magnitude less axonal currents than the worm axon. APMF estimates of mammalian neurons reported from computational studies are inconsistent and vary in the range ~1 pT–1 nT^28,40,41^. Further, the contribution of currents in the different types of axonal segments, like axon hillock, nodes of Ranvier, and others, to the final APMF has not been investigated. In an intact mammalian brain, detecting activity based on APMFs from axonal currents requires localization of the source at single-neuron resolution. Only 2D measurements of the magnetic field via widefield ensemble NVC magnetometry would not be usable, as the 3D source reconstruction would be non-unique. We first address the question—are there specific signatures in the APMF of a neuron that can allow spatiotemporal reconstruction of the source? For this purpose, we consider a realistic cortical pyramidal neuron APMF and investigate the differential contribution of neuronal regions to the APMF magnitude.

**Fig. 1:**
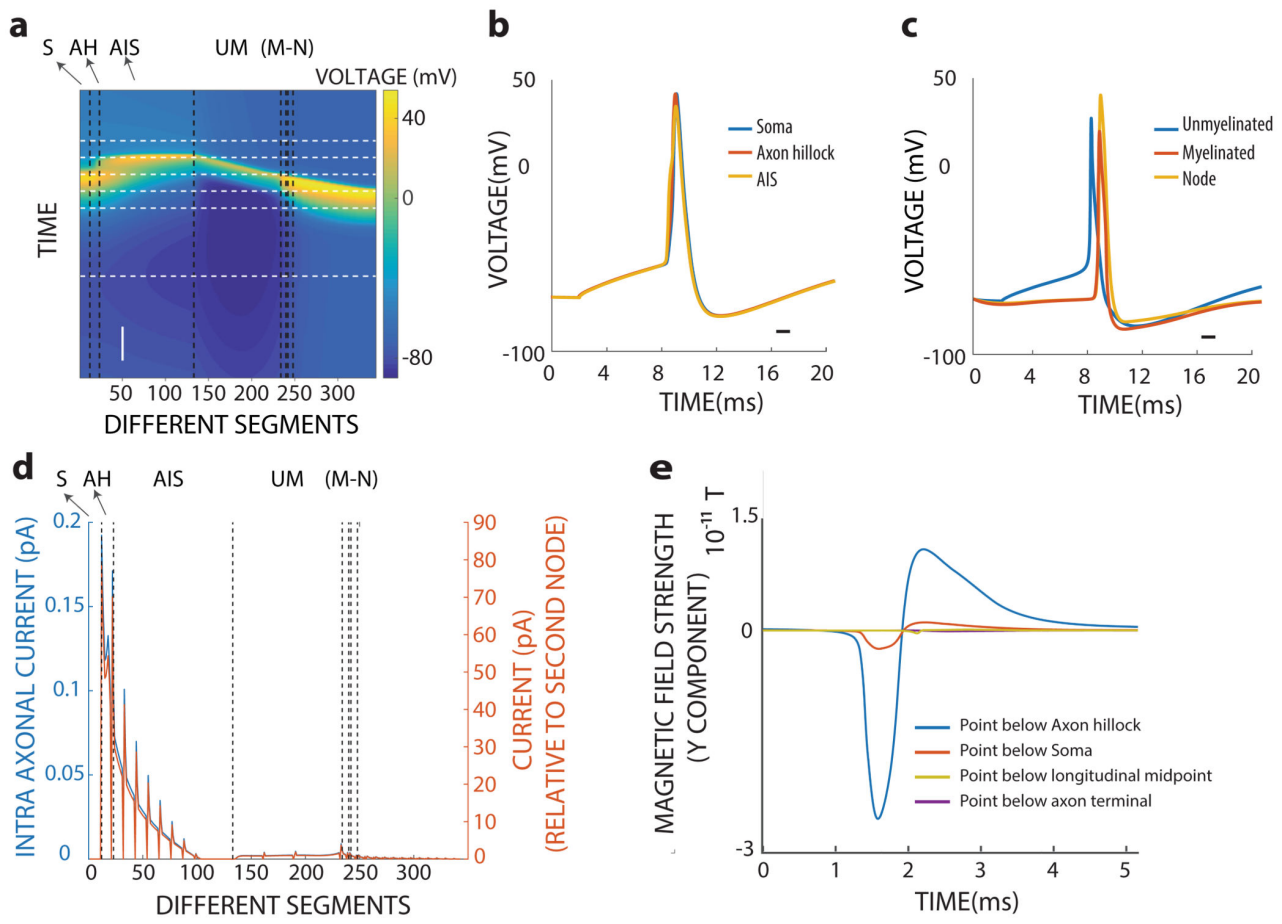
Simulations of membrane potential and estimation of intra-axonal currents **a** Color map of membrane potential as a function of time and spatial segments of the neuron. Dashed vertical lines (black) mark the boundaries of different neuronal regions on the map. Alphabet codes denote the names of neuronal segments. S soma, AH axon hillock, AIS axon initial segment, UM unmyelinated region, M-N myelinated region–node of Ranvier repeating one after another. Only the first two myelin node boundaries are marked by vertical lines for clarity in the figure. The action potential (AP) originates in the most distal end of AIS region and propagates in a bidirectional manner. Horizontal white lines denote timepoints 2.5, 3, 3.5, 4, 4.5, and 6.5 ms, respectively, considered in further analysis. Scale bar denotes 1 ms. **b** Membrane potential profile of particular segments of soma (blue), axon hillock (red), and AIS (yellow). Membrane potentials for these three segments are nearly equal. The scale bar denotes 1 ms. **c** Other membrane potential profiles of particular segments of the unmyelinated axon (blue), myelinated region (red), and node of Ranvier (yellow). The scale bar denotes 1 ms. **d** Peak intra-axonal current profile of different neuronal segments. Left axis: Maximum intra-axonal current that flows through the most proximal segment of axon hillock. Right axis: Peak intra-axonal current profile normalized by peak intra-axonal current in the second node of Ranvier. Peak currents in axon hillock are nearly two orders larger than other axonal regions. **e** Y component of magnetic field measured at a point perpendicularly below different segments of the neuron has been shown. Measurement points below axon hillock (blue), soma (red), longitudinal midpoint (yellow), and axon terminal (purple) have been compared. The magnetic field at point perpendicularly below axon hillock is significantly larger as compared to magnetic fields at other points, denoting the dominance of axon-hillock contribution to the magnetic field. Magnetic field traces of point below the longitudinal midpoint and axon terminal are close to zero on this relative scale.

The voltage propagation through the structures in a realistic cortical pyramidal neuron model^35^ was simulated to study the contribution of the distinct types of sodium channels in the initiation of the AP. The model incorporated realistic geometry, physiological parameters, and experimentally determined ion-channel densities. The pyramidal neuron comprised of different segments namely, cell soma, dendrites, axon hillock, action initial segment, unmyelinated axon, myelinated axon, and nodes of Ranvier. The neuron was divided into isopotential compartments, and membrane potential dynamics across these compartments was governed by the cable theory^42–44^ equation (Eq. (3), “Methods”), and solved using the NEURON solver^45^ (see “Methods”). A step current injection was added at “central” soma segments to make the neuron fire APs, and the membrane potential for one AP was recorded. Voltage propagation across each segment in time for a single AP was imported from NEURON^41,45^, and all further analyses were done in MATLAB^46^. AP initiation was observed in the most distal segment of the axon initial segment (AIS) (Fig. 1a, AIS region). This result is consistent with previous studies, which show that AP originates in the AIS, due to the presence of high density of sodium channels and high resistance of segments^35^. After AP initiation, a bidirectional propagation of AP, one in the direction of cell soma and the other in the direction of axon, is observed (Fig. 1a–c). The bi-directionality is in agreement with observations from experiments^35^. In order to estimate the mammalian neuron’s APMF, intra-axonal currents across segments at each time instant were calculated. Only the intra-axonal currents were considered in calculating the APMF based on previous theoretical work^33,47,48^ showing that the net magnetic field due to spiking in neurons is primarily determined by the intra-axonal currents. This assumption has also been experimentally demonstrated in superconducting quantum interference device (SQUID)-based magnetic field measurements of frog sciatic nerve^49,50^. The intra-axonal current profiles in each segment (Fig. 1d) were calculated (Eq. (4)), “Methods”) based on the discrete version of the basic cable equation. We observe high intra-axonal current flow in the axon-hillock region (Fig. 1d). Further, we analyzed intra-axonal current flow in all regions of the neuron, as a fraction of the current flow in the second node of Ranvier (Fig. 1d). The discontinuities observed in the curves of Fig. 1d are inherent to numerical solutions of the pyramidal neuron model, occurring specifically at compartments where radius, physiological type, or other properties of any segment changes (see Supplementary Note 1 for detailed description). We found two orders of magnitude higher current flow in the most proximal segment of the axon hillock as compared to the second node of Ranvier. This result implies that pixels on the NVC sensor that are on the perpendicular axis of the axon hillock will sense significantly higher magnitudes of APMF signatures. Also, the presence of ion channels in segments of neurons leads to current injection in segments. Higher currents will be found in segments with relatively high sodium ion-channel density and large surface area. While both Axon hillock and AIS have high sodium channel density, axon hillock has a much larger surface area, especially toward the proximal end connecting to the soma. This provides a physiological explanation to higher axonal currents in axon hillock.

Comparisons of APMF magnitude and waveforms (calculated by Eq. (5), “Methods”) between a point located vertically below the axon hillock to a point located vertically below the soma or axon terminal clearly implicate the axon hillock as the dominant contributor to the APMF (Fig. 1e). We quantify the mammalian APMF magnitude as the peak-to-peak magnetic field (Y component) measured at a point vertically below the axon hillock, which is 36 pT at a distance of 20.50 μm from the longitudinal axis passing through the centre of the axon. However, no experimental verification of the mammalian neuron APMF magnitude has been made yet to the best of our knowledge.

We have excluded the magnetic field contribution of soma and dendrites from the analysis. It has been shown analytically that the magnetic field contribution due to spherical soma will be zero^51^. Pyramidal soma shape might generate an effective magnetic field due to distortion from the spherical shape. We assume it to be small due to the relatively smaller sodium ion-channel density of the soma. Therefore, we have excluded somatic contribution in magnetic field calculations. Also, magnetic field contribution from dendritic compartments, due to small diameters and relatively low sodium ion-channel densities, have also been excluded.

### 2D NVMM comprises of specific signatures of APMF

**Fig. 2:**
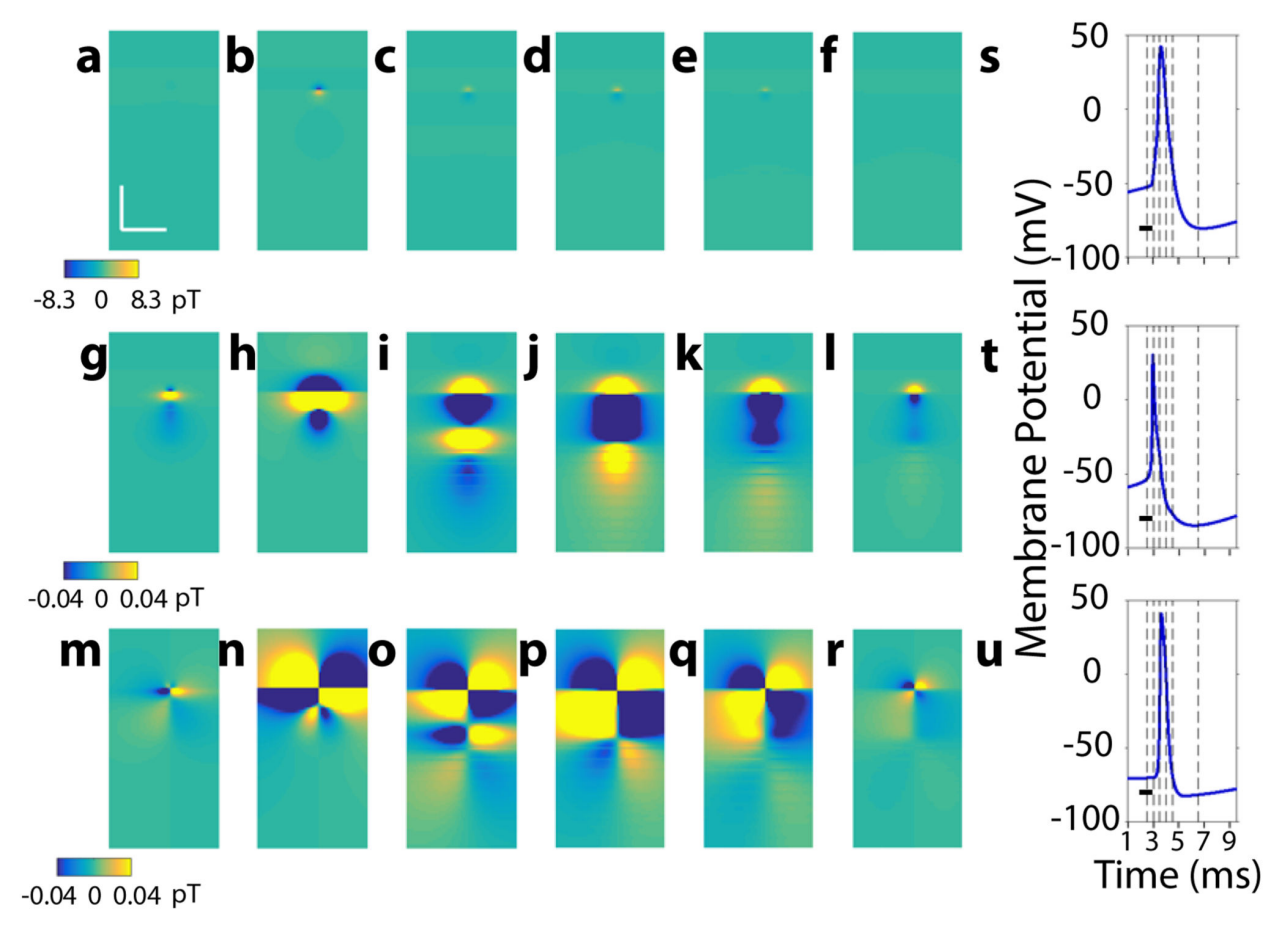
2D diamond-nitrogen-vacancy magnetometric maps (NVMM) of a single mammalian action potential 2D NVMMs are shown at specific timepoints, in the order 2.5, 3, 3.5, 4, 4.5, and 6.5 milliseconds (ms) relative to a reference time zero where the membrane potential is below the firing threshold. **a-f** 2D NVMMs of the Y component of the magnetic field at timepoints specified in above order. Action potential-associated magnetic field (APMF) signatures are prominent only at timepoints corresponding to action potential (AP) passing mainly through the axon-hillock region, as observed in panels **b-e.** Color axis marks Y magnetic field magnitude and is limited to the maximum magnitude of the magnetic field across all timepoints, 8.3 pT for these NVMMs set. Both orthogonal scale bars denote 400 μms. **g-l** 2D NVMMs of the Y component of the magnetic field, with a reduced saturated color axis (+/−0.04pT) to show other weak APMF patterns in these NVMMs, presented in the same time order as before. Weak APMF patterns, like AP passing through repeated regions of myelinated region and node of Ranvier, are not visible when scaled to the maximum magnetic field due to relative dominance of axon-hillock contribution. The spatial scale bar is the same as that of panels **(a-f). m-r** 2D NVMMs of the Z component of the magnetic field, with a saturated color axis (+/−0.04 pT) to show other weak APMF patterns in these NVMMs, presented in the same time order as before. The spatial scale bar is the same as that of panels **a-f. s-u** Plots to understand mapping of 2D NVMMs at different timepoints to membrane potential of specific neuronal segments. Temporal membrane potential profiles of (**s**) axon-hillock proximal end, (**t**) axon initial segment (AIS) middle segment, and (**u**) first node of Ranvier have been shown with timepoints marked by vertical dashed lines in the same order as 2.5, 3, 3.5, 4, 4.5, and 6.5 ms. Scale bars in all three plots correspond to 1 ms. Panels **s-u** share same *x* axis, as labeled for (**u**).

We generated 2D time-varying magnetic field maps by spatially summing magnetic field contributions from current flow in different segments of the neuron, at each time instance (Eq. (5), “Methods”). We explain the features in these maps with respect to the following: bidirectional propagation of AP, the activity in nodes of Ranvier, and the overall spatial size of APMF signatures. These 2D NVMMs are simulated realizations of magnetic field measurements on a 2D plane, as it would be in an experimental case of a thin top layer of NVC defects in a cube of the diamond (see “Methods”). Henceforth, we refer to the above 2D NVMMs simply as maps, unless mentioned otherwise. The simulated maps at different timepoints (Fig. 1a, horizontal lines) acquired during the firing of an AP show a number of variations of features (Fig. 2). Here, we show noiseless 2D maps at different timepoints, namely, 2.5, 3, 3.5, 4, 4.5, and 6.5 ms in Fig. 2a–f, respectively, to understand fundamental features of NVMMs and correlate it with AP propagation in neurons. The first prominent feature in the maps is the dominance of the axon hillock, as we observed that APMF signatures are significantly visible in only maps of Fig. 2b–e, which corresponds to AP propagating through or near the axon-hillock region of the neuron. Since the axon-hillock activity’s contribution to the APMF was approximately two orders larger than those of other regions APMF, signatures of other regions were unidentifiable in these maps. Therefore, to understand the APMF signatures of other regions in these maps, we saturate the color axis at lower magnetic fields and separately analyze the **B**_y_ (Fig. 2g–l) and **B**_z_ (Fig. 2m–r)) component of the magnetic field. Each map (Fig. 2a–r) corresponds to AP propagating through specific segments (Fig. 2s–u) and at specific timepoints (dashed vertical lines, Fig. 2s–u). The membrane potential of the three example segments (Fig. 2s—axon hillock, 2t —AIS, and 2u—node of Ranvier) show that the AP initiation falls before timepoint 3 ms. After initiation of the AP, we observed bidirectional propagation of AP along the cell soma and axon terminals (Figs. 1a and 2a–r). Later in time (4–6 ms, after the start of current injection), we observed repetitive patterns of activity that correspond to AP propagation through repetitions of myelin node in the neuron (Fig. 2j–l, p–r). Another important feature was the appearance of quadrant like **B**_z_ components of the field (Fig. 2m–r), which indicates that intra-axonal currents can be approximated as current dipoles.

### Reconstruction as a dictionary-based linear inverse problem

The inverse problem comprises detecting the time and location of neurons that fired an AP from NVC maps. The magnetic field, as measured by ensemble diamond magnetometry, due to current flow across segments of spiking neurons is given by Eq. (1)

(1) $$B_{\text{nv}}\left(\alpha ,\beta ,t\right)=\sum_{n}kI\left(x,y,z,t\right)\frac{\vec{dl}\;X\;\vec{dr}}{{\left|\vec{r}\right|}^{2}}.$$

In the above equation, **B**_nv_(*α*, *β*, *t*) is the field experience by an NVC or a small ensemble of NVCs located in the pixel at position (*α*, *β*) at time *t*, *n* denotes all isopotential segments (of all neurons), *k* is a constant (“Methods”). The inverse problem is to detect a fraction of *I*(*x*, *y*, *z*, *t*) waveform that guarantees an AP in neuronal soma at location *x*, *y*, *z*, and time *t*, by operating on the vector magnetic field **B**_nv_(*α*, *β*, *t*) from different diamond NVC pixels obtained from diamond NVC vector magnetometry. It is to be noted that, since an AP is an all or none event, we do not need to reconstruct the full spatiotemporal variation of current *I*.

The above inverse problem can be formulated in terms of a dictionary-based linear inverse problem, where the dictionary elements contain prior information about NVMMs from AP firing of single neurons located at different spatial locations in different orientations. Since the axon-hillock currents provide the dominant signature in a neuron’s APMF, the individual dictionary elements are created by considering mainly the axon-hillock NVMMs (Fig. 2c–e). The above linear representation allows the application of a matching pursuit algorithm for spatiotemporal AP reconstruction^37,38,52,36^. A dictionary-based matching pursuit approach is motivated by sparse spatiotemporal distribution of spikes in mammalian cortices^52–54^ and previous application of matching pursuit algorithms to magnetoencephalography (MEG)/electroencephalography (EEG) data^55^ for the reconstruction of active current dipoles formed during APs. However, reconstruction of MEG/EEG has been demonstrated only at the coarse spatial resolution, in the range of hundreds of microns, not near the single-cell spiking resolution.

The final experimental map is expressed as a linear combination of individual NVMMs. For the dictionary matrix *A*, *X* a vector of length equal to the number of neurons and *B* a vector of experimental multidimensional NVMM data points, we can write the problem as in Eq. (2), in which *X*, a binary vector, needs to be estimated.

(2) AX=B+ϵ.

The dictionary *A* is of *mxn* dimensions, where *m* is the number of dimensions in the experimental data, *ϵ* is the noise in NVMM experimental maps, and *n* is the total number of neurons in the tissue volume of interest. We aim to solve for *X*, whose elements can only be zero or one, depending on whether the corresponding neuron fired or not. However, the above linear equation is overdetermined, with the number of neurons being less than the number of dimensions in an NVMM. Performing a full least-square search for 2^*n*^, where *n* is in thousand range, is computationally impractical, and hence the following dictionary-based matching pursuit algorithm is used after modification for NVMM time series data.

### Proposed matching pursuit algorithm

**Fig. 3:**
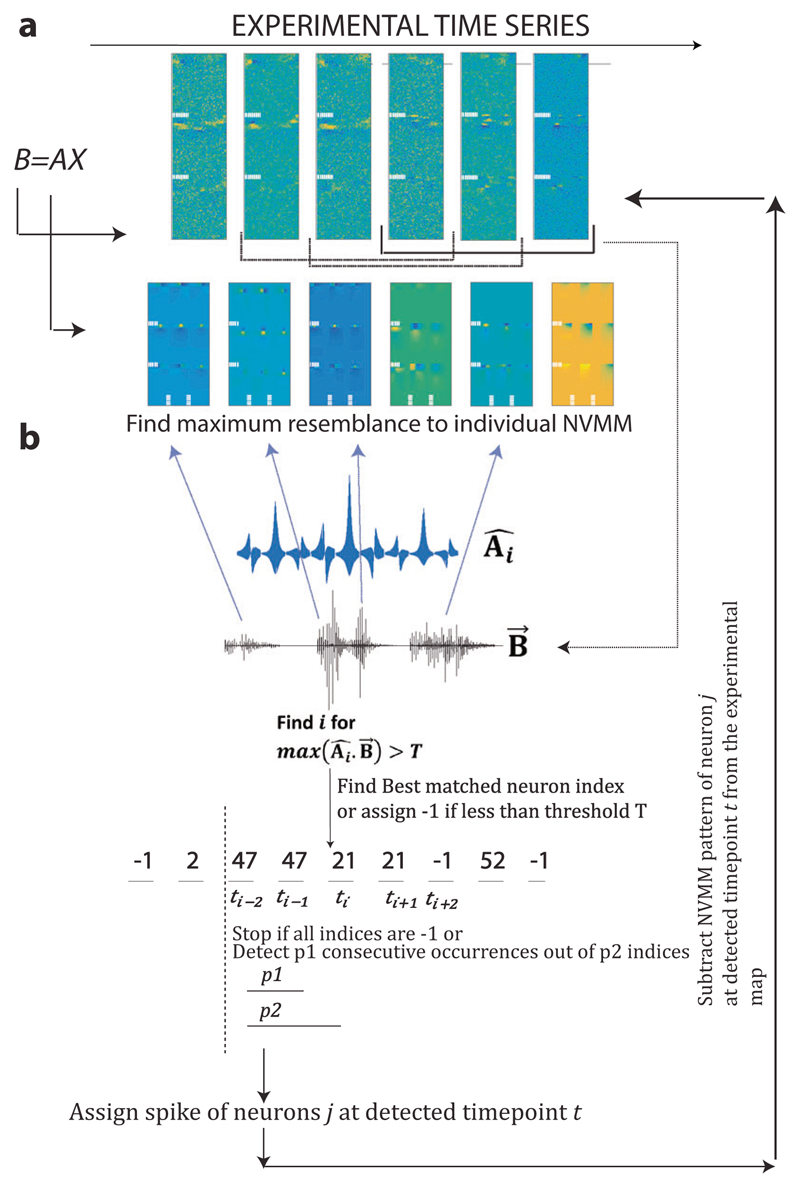
Schematic of the reconstruction algorithm for detecting action potential timing and location from 2D diamond–nitrogen-vacancy vector magnetometric maps (NVMMs) The algorithm is recursive in nature, and the inverse problem has been cast into a linear framework of *B* =*AX*. In each step, a set of experimental time series of NVMMs is given as input. **a** An example experimental time series maps (**B**) and example dictionary maps (**A**) are shown. Three successive timepoints of the time series are concatenated (three such successive groups are marked at the end) to be used further. At each timepoint, the experimental 2D map consists of three maps corresponding to X, Y, and Z components of the magnetic field (separated by dashed white horizontal lines). Dictionary elements are also set up accordingly with three specific timepoints of the action potential (AP) and with three orthogonal XYZ magnetic field values. **b** All 2D maps and dictionary elements are converted to 1D signals, shown as 1D vectors in the schematic. **Â**_*i*_ ; **B→**(*t*) corresponds to 1D vector of ith element of the dictionary and 1D vector of the experimental map containing vector magnetic field value at time *t*, respectively. Each experimental timepoint is matched with all dictionary elements, and the resemblance is determined by a dot product. *T* denotes a threshold value for the dot product. Angled Arrows from experimental time series B→(t) to example maps of dictionary symbolize different combinations of neuronal signatures are present in the experimental map. Series of best-matched neuron indices are processed further to look for consecutive occurrences of the same neuron and transitions from one neuron to the next. Best-matched neuron indices are marked by *t*_*i*_, where *i* varies for different discretized timepoints. *p*1 and *p*2 are algorithm parameters, denoting search for *p*1 consecutive occurrences of a best-matched neuron index out of *p*2 consecutive timepoint indices (*t*_*i*_). Based on the search criterion, NVMM signals of selected AP instances (space and time of AP) are subtracted from the experimental maps, and residuals are equated to experimental maps for the next iterations. The algorithm stops when no relevant AP signature is detected in experimental maps.

The proposed reconstruction algorithm (details in “Methods”) works by considering a multidimensional time series **B**_*t*_ considering each pixel in maps of **B**_x_, **B**_y_, and **B**_z_ at each of three successive timepoints (*t* − 2, *t* −1, *t* for *n*_t_p__ = 3 in “Methods”, Fig. 3a) as a dimension. Each of the **B**_x_, **B**_y_, and **B**_z_ 2D maps (image data representing each timepoint) in the time series are represented in Fig. 3a separated by white dashed lines. At each time instant, the signal acquired along with the signal in the previous two time frames can be projected onto individual normalized columns **Â**_*i*_, of the dictionary *A* (Fig. 3b). The maximum projection, and hence, the most probable single neuron that fired an AP at that instant is denoted by the best-matched neuron index in schematic Fig. 3. We impose the condition of detecting the spikes of a particular neuron as occurrences of the same neuron as the best-matched neuron at multiple time instances, greater than a specified parameter *p*1 within a stretch of successive *p*2 timepoints. On detecting a spike, we ascertain the exact spike timing of the neuron by matching the neuron’s spike signal to different regions of the experimental signal with a local shift of +/−*τ* timepoints (1.5 ms total) near the timepoint where we detected neuronal spike. The time instant that gave maximum dot product/alignment with the neuron’s signal was chosen at the exact spike timing of the particular neuron. Since axon-hillock activity is the dominant signature on NVMMs, each element in the dictionary is constructed from additions of three timepoints 3.5, 4.0, and 4.5 ms, which mainly correspond to high axon hillock activity (Fig. 3b). The main control parameters of the reconstruction algorithm are threshold *T*, *p*1, and *p*2. The threshold needs to be set greater than the smallest energy column of the dictionary. *p*2 parameter depends on the total number of timepoints considered in the formation of the dictionary. *p*1 controls the minimum number of consecutive timepoints that a neuron should be best matched to the signal to be considered a spiking event. For later demonstrations of reconstructions, we considered *p*2 equal to 3, which is equivalent to considering 3.5, 4, and 4.5 ms time-point NVMMs being incorporated into the dictionary. *p*1 has been taken to be 2, equivalent to a millisecond length signal in real time.

We developed this algorithm as a modification of the original matching pursuit algorithm. Our choice of maximum dot product (DP) criteria (“Methods”) was based on the use of these criteria in previous matching pursuit algorithms. These algorithms are based on the assumption that the experimental data can be expanded as linear combinations of basis vectors, which are individual dictionary elements. Hence, dot products with basis vectors are commonly used to extract coefficients. Another similar criterion to evaluate the resemblance between experimental time series and dictionary elements is Pearson’s correlation coefficient (CC). By changing the best-matched index selection criteria from DP to CC (“Methods”), we found identical performance in 2D case and minor differences in 3D case (see Supplementary Table 3).

### Population performance in cortical tissue simulations

**Fig. 4:**
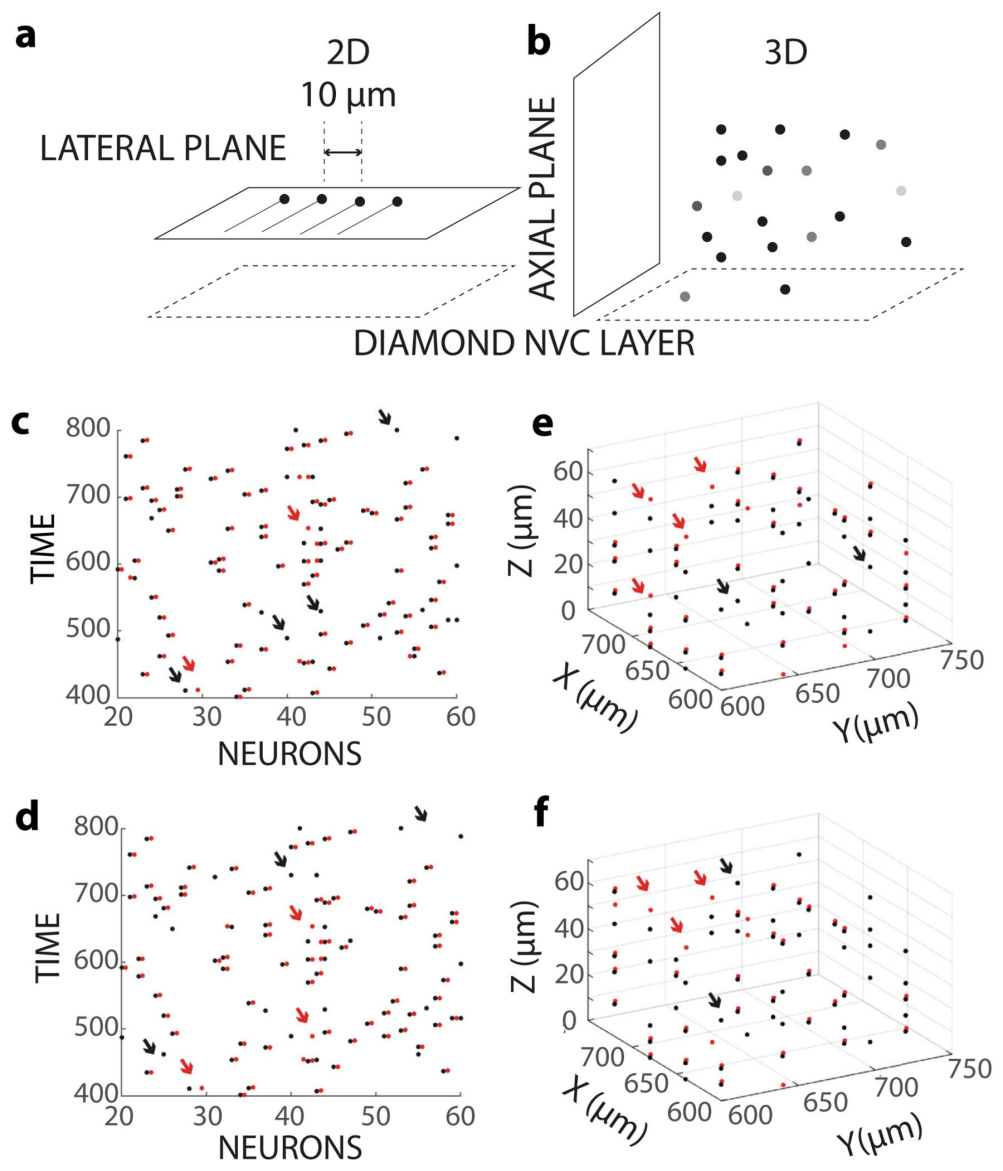
Population reconstruction performance in neuronal 2D layer and 3D volume **a** Schematic showing arrangement of neurons in the 2D layer in a plane parallel to the diamond–nitrogen-vacancy-center (NVC) layer. **b** Schematic showing the arrangement of neurons in a 3D volume placed above the diamond NVC layer. **c, d** Visualization of performance of the algorithm, for a 2D case without noise (**c**) and with added Gaussian experimental noise (**d**), where black dots are actual action potential (AP) instances and red dot show AP instances marked by the algorithm. x axis denotes the lateral position of the 2D neuron, and y axis denotes time (0.5 ms per unit). Only few neurons are shown in this figure for clarity. For correct reconstruction, red and black dots overlap, but have been slightly shifted to compare reconstruction. Examples of false positives are marked with red arrows, and examples of missing correct cases have been marked by black arrows. **e, f** Visualization of the performance of the algorithm for a 3D case without noise (**e**) and with added Gaussian experimental noise (**f**). Representation of AP instances, correct detection, false positives, and misses are same as that of 2D case (**c, d**). All actual/reconstructed events have been integrated in time into one 3D plot, where the three dimensions correspond to the cell soma location of neurons. Only few neurons have been shown for the 3D case, similar to the 2d case, in this figure for clarity.

The feasibility of spatiotemporal localization of occurrence of spikes based on the APMF of the axon-hillock current with our algorithm was tested in the case of a 2D array of neurons and a 3D volume of neurons (Fig. 4a, b). We quantify the performance of the algorithm in reconstructing spike location and time in each case. In both cases, spikes in neurons at each time step, of 500 μs, were generated as a binomial process with a probability *f* (see “Methods” for details). In the 2D case, neurons are placed in the plane parallel to the diamond NVC layer, at a spacing of near-cell soma size separation of 10 μm, and spikes are assigned as described in “Methods”. Experimental NVMMs are generated by the summing of individual NVMMs and adding noise (Gaussian or shot noise). Figure 4c illustrates an example of reconstruction where we observed that most of the spikes are detected and marked correctly in space and time (Fig. 4c).

The performance of the algorithm for proper detection of spikes in space and time can be obtained with *d*′ = *z*(hitrate)–*z*(false alarm)^56^. The algorithm is very robust in the sense that it correctly rejects the absence of spikes even in large noise, thus leading to a very high correct rejection rate or very low false alarm. Given the extremely low percentage of false alarms (≪1%) with the naturalistic sparse firing rates considered, the *d*′ value is very large in most cases even with noise. Hence, to be conservative, we quantify the accuracy of reconstruction based on the fraction of correctly marked spikes by total spike instances marked by the algorithm. This would mimic a real situation when actual imaging is performed in the absence of knowledge of all possible spikes that could occur. In the no noise case, the performance was 83.61 +/− 2.17% (Fig. 4c), and in the case of added Gaussian noise corresponding to a signal-to-noise ratio (SNR) of − 11.8736 dB (see “Methods” for SNR calculations), the performance was 83.82 +/− 2.09% (Fig. 4d). Here, we conclude that the matching pursuit algorithm can have inherent errors in reconstruction, even without noise, but the reconstruction shows high resilience to Gaussian noise.

The 3D setup is a more complex case, as NVMM of a single neuron closely resembles not only neurons in the same lateral plane but also nearby neurons in multiple directions. Further, due to varying distance from the diamond NVC layer, neurons in the different axial planes have varying magnitudes of NVMMs. We show that such an ensemble of neurons can be reliably reconstructed (Fig. 4e, f). We found performances of 68.77% (+/−1.41) without noise (Fig. 4e) and 71.7281% (+/−1.1886) with Gaussian noise (Fig. 4f). Similar to the 2D case, 3D reconstruction is resilient to Gaussian noise at SNR − 9.46 dB. However, a higher SNR ratio is needed to perform reconstruction in a 3D setup due to a more complex correlation structure in the columns of the dictionary. The overall detailed results of performance for the 2D and 3D cases, with and without noise, are provided in Supplementary Table 1.

To compare noise resilience in another method, we performed Moore–Penrose pseudoinverse^57^-based reconstruction of the same linear inverse problem in 3D setup (Supplementary Fig. 1). In pseudoinverse-based reconstruction, without noise reconstruction is 96.19%, but it shows high sensitivity to noise, as the performance drops to 20% and a very low fraction of detected spike instances. Due to high correlations in pairs of columns of the dictionary representing closely spaced neurons, the dictionary is an ill-conditioned matrix, and hence pseudoinverse solutions are highly sensitive to noise.

Based on the magnitude of the actual magnetic field, ideally, timepoint 3.0, 3.5, and 4.0 ms NVMMs should be used as the dictionary elements. The above frames contain the highest magnetic field signatures and are closest to the axon-hillock activity. Hence, initially, we performed 2D and 3D reconstruction simulations with these timepoints. Supplementary Table 2 contains the details of performance with the above timepoints (3, 3.5, and 4 ms) constituting the dictionary elements. While the 2D reconstruction was decent, the 3D reconstruction was not satisfactory. A critical difference in these set of time frames is the opposite directions of magnetic field signatures between NVMMs at 3 and 3.5 ms (Fig. 2). We believe that the algorithm, primarily in the 3D case, is sensitive to this directionality of the magnetic field (see Supplementary Note 2 for further discussion).

### Resolvability of spatially and temporally close distinct APs

The most important issue in reconstruction is the resolvability of spikes in nearby neurons (space) and time. The limits of resolvability or resolution of reconstruction would determine if single-cell resolution imaging can be performed with the proposed technique and algorithm. We analyze whether only two nearby neurons, separated by distances comparable to single soma size, ~10–20 μm, can be reconstructed by the algorithm. In the entire population of the 2D or 3D cases, only two nearby neurons are assigned one spike, each with a fixed time difference of Δ*t*. Experimental time series for these two spike events are formed, and the algorithm is applied to reconstruct the spike times and location. Initially, no noise is added to the experimental time series. Also, the neurons are in the same geometric arrangement as shown for performance reconstruction (Fig. 4a, b).

**Fig. 5:**
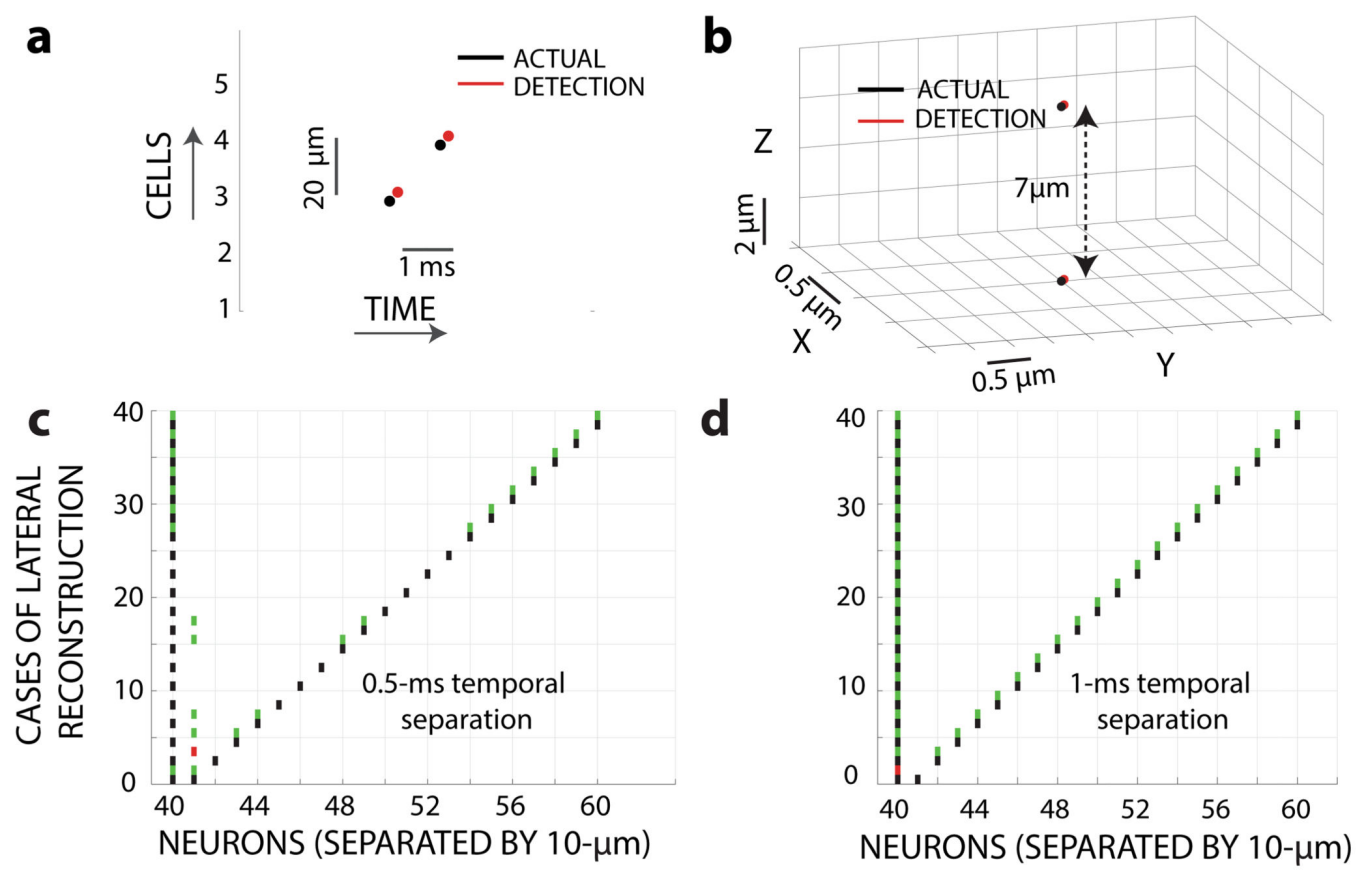
Single-cell spatial resolution by reconstruction from Gaussian noise dominated 2D diamond–nitrogen-vacancy vector magnetometric maps (NVMM) **a** Plot showing two laterally separated neurons (both neurons located in the plane parallel to diamond–nitrogen-vacancy-center (NVC) layer separated by 20 μm, firing action potentials (APs) near simultaneously, at one millisecond difference. Actual AP timings and location have been marked with black dots. Reconstruction of AP instances (timing and location) from the proposed algorithm has been marked by red dots. The dots coincide but have been slightly shifted for visualization. **b** Plot showing two axially separated neurons (neurons separated along plane perpendicular to diamond NVC layer) separated by 7 μm and 0.5 ms AP timing difference. Color code same as panel (**a**). **c** Plot showing case by case reconstruction accuracy (20 cases X2, actual and algorithm) with varying lateral separation (per neuron number, 10 μm shift) between two neurons located in the same diamond NVC plane. AP timing difference fixed at 0.5 ms. Black vertical lines show the location of actual spiking neurons. Green vertical lines show spike instances marked by algorithm, if spike times are detected correctly. Red vertical lines show spike instances marked by algorithm, if spike times are incorrect. **d** Plot showing case by case reconstruction accuracy (20 cases X2, actual and algorithm) with varying lateral separation (per neuron number, 10 μm shift) between two neurons located in the same diamond NVC plane. Actual AP timing difference fixed at 1 ms. Color code and y axis same as of panel **c**.

Single best-reconstruction cases are shown for the 2D (Fig. 5a) and the 3D (Fig. 5b) cases. In the 2D case, cells separated by 20 μm and with spike-time differences of Δ*t* = 1ms are correctly reconstructed. Similarly, in a 3D case, an axial pair of neurons separated by 7 μm and with the spike time difference of Δ*t* = 0.5 ms are accurately reconstructed.

To better understand the resolvability of nearby cells by the algorithm, we successively vary the lateral separation, the distance between two parallel neurons in the same plane (2D case), from 200 μm to 10 μm in steps of 10 μm with fixed Δ*t*. These individual 20 experimental time series, with no noise added, form 20 different cases of reconstruction. Two different spike-time differences of Δ*t* = 0.5 ms and Δ*t* = 1ms have been studied and represented in Fig. 5c, d, respectively. For each separation case, the locations of spiking neurons are marked by black vertical ticks. Cases of reconstruction where the spike timings of spike instances marked by the algorithm exactly match the spike timings of the actual case are shown by green ticks below the corresponding black ticks. Cases of reconstruction, where the algorithm marked an incorrect spike are shown with red ticks. Cases of reconstruction where the algorithm did not mark any spike instance are empty.

For Δ*t* = 0.5 ms (Fig. 5c), we observe neurons separated by more than 130 μm are correctly reconstructed. However, when spiking neurons are brought closer in space, mostly spike instances are not marked by the algorithm or incorrectly marked by the algorithm. Nearby neurons have highly correlated dictionary elements, and experimental timepoint maps where activity from both neurons are present can resemble some other nearby neurons. Therefore, continuous stretches of p1 indices are not formed (see “Methods”—Algorithm), and hence, no spike instance or incorrect spike instances are marked by the algorithm. However, for one millisecond time difference cases (Fig. 5d), we observe correct reconstruction till the minimum separation of 20 μm. The lateral resolution in one millisecond case improves due to a lesser temporal overlap of NVMM signals of nearby neurons.

Further, we test the extent to which Gaussian noise can affect the above reconstruction cases of nearby neurons. The above reconstruction of two nearby neurons becomes stochastic by the addition of Gaussian noise to the experimental time series. Therefore, a minimum SNR is required for the algorithm to perform correct reconstructions, and a lower minimum SNR is an indicator of high resilience of reconstruction to Gaussian noise.

To estimate minimum SNR for the 2D (lateral separation) and 3D (axial separation) case, we perform multiple repetitions of reconstruction of two nearby neurons, as above (Fig. 5) but with added Gaussian noise to the experimental NVMM. To consider the worst-case scenario, the accuracy of reconstruction has been studied in the 2D case, for one spike each from two nearby neurons which are laterally separated by 10 μms and Δ*t* = 0.5 ms. For the 3D case, one spike each from two nearby neurons which are axially separated by 7 μm and Δ*t* = 0.5 ms. By varying levels of Gaussian noise factor, we vary SNR and simulate reconstruction (run algorithm) for 50 independent repetitions of the two-neuron case for both 2D and 3D. The minimum SNR is considered as a point where the standard deviation of reconstruction drops to zero (for these 50 repetitions). For each repetition, a correct reconstruction, where algorithm marks correctly the neuron and its spike time both, is given value 1 and 0 is given otherwise.

**Fig. 6:**
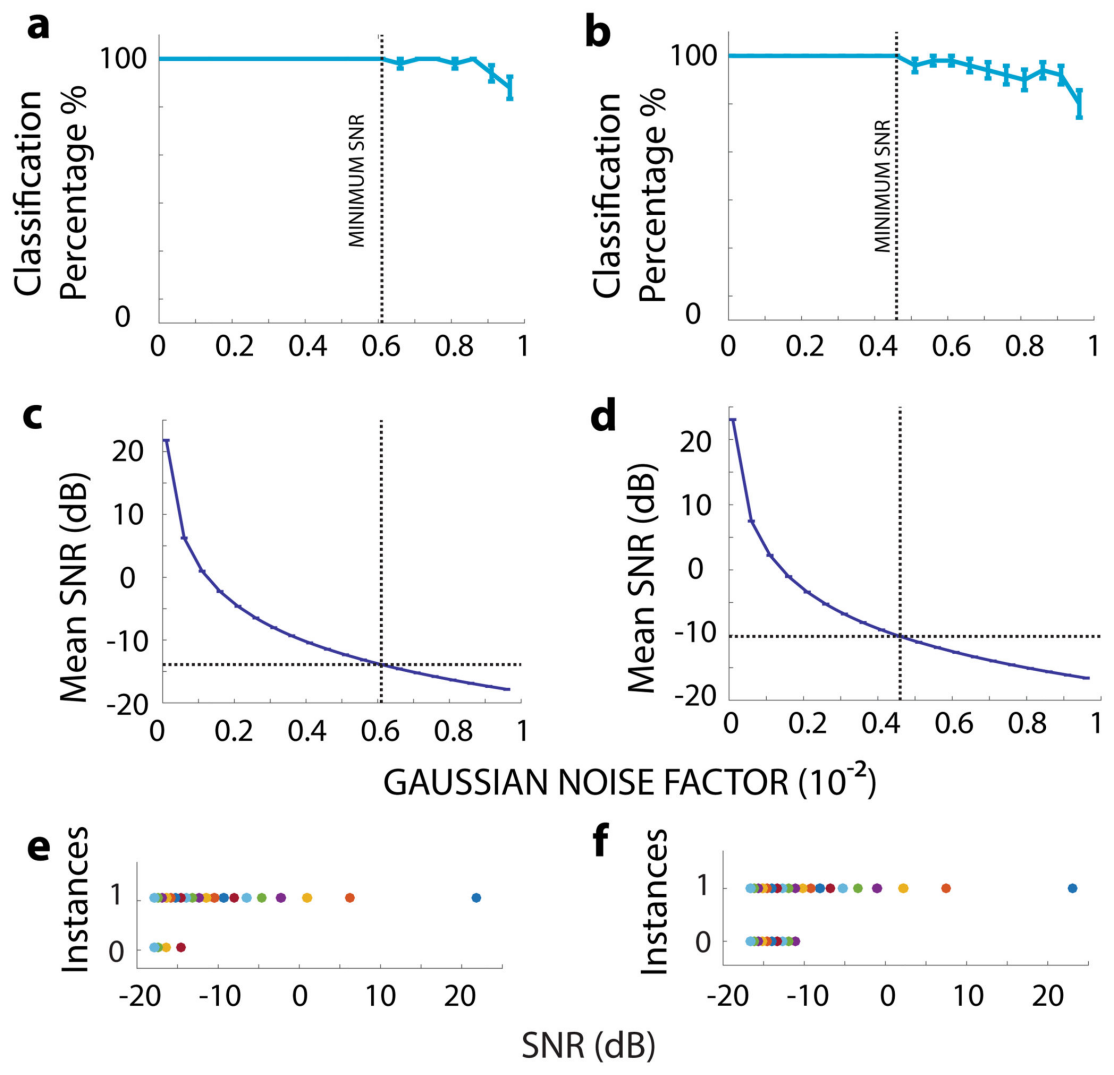
Resolvability of spatially and temporally close action potentials at varying Gaussian noise levels Minimum required signal-to-noise ratio (Min. SNR) is shown, for resolving action potentials (APs) from laterally and axially separated nearby neurons from 2D diamond–nitrogen-vacancy-center magnetometric maps (NVMMs) added with Gaussian noise. **a** Plot of correct classification percentage versus Gaussian noise factor, a factor to control levels of Gaussian noise added to NVMMs, for lateral separation case (two neurons, 10-μm apart, spike-time difference 0.5 ms). Min. SNR has been marked as the point where the standard deviation (standard error bars shown) of the correct classification percentage drops to zero (in 50 repetitions). **b** Plot of current classification percentage versus Gaussian noise factor for axial separation case (two neurons, 7-μm apart, spike-time difference 0.5 ms). The vertical line marks the point of Min. SNR. **c** Plot of mean SNR (dB) versus Gaussian noise factor for lateral separation case. **d** Plot of mean SNR (dB) versus Gaussian noise factor for axial separation case. Panels **a–d** plots share the same x axis title–Gaussian noise factor. **e, f** Example individual instances of correct (1)/incorrect (0) reconstruction by proposed algorithm versus SNR (dB) for lateral separation case (**e**) and for axial separation case (**f**). A clear shift in certainty of reconstruction is observed at SNR above Min. SNR. Also, colors where randomly assigned for dots corresponding to different SNR levels and individual dots might be further separated on SNR axis at finer scale, as different trials with same Gaussian noise factor generate slightly varied SNR All error bars in this figure represent one standard error over 50 independent trials. For each value of Gaussian noise factor, 50 independent NVMMs were produced, and correct/incorrect classification was performed for each of these NVMMs by the proposed algorithm.

We observe decreasing standard error and increasing mean of correct classification percentage (average of individual 0/1 values) to 1 as the Gaussian noise factor decreases for 2D case (Fig. 6a). Similar trends are observed for the 3D case as well (Fig. 6b). The point of minimum SNR is marked as a dashed vertical line for both the 2D and 3D cases in Fig. 6a, b. Since the Gaussian noise factor is not linearly related to SNR (see the section “SNR calculations”), the actual mapping between them is shown in Fig. 6c for 2D case and Fig. 6d for the 3D case. We find -13.9 dB minimum SNR for the 2D case (at Gaussian noise factor = 0.0061) and −10.2 dB minimum SNR (at Gaussian noise factor = 0.0046) for the 3D case. A higher minimum SNR required for the axial case is due to more complex correlations of any single neuronal NVMM to other neuronal NVMMs in nearby volume as compared to a lesser number of nearby correlated neuronal NVMMs in a plane in the lateral case. We observed a clear certainty in individual events of reconstruction (for 50 repetitions) when the SNR is higher than the minimum SNR for both the lateral (Fig. 6e) and axial case (Fig. 6f).

A negative minimum SNR shows the high resilience of the proposed algorithm to Gaussian noise. Further, it implies that reconstruction might be possible in diamond NVC experiments with lower magnetic field sensitivity, where the noisy magnetic field data can be compensated with prior information based on the axon-hillock’s APMF signature in the dictionary.

While the experimental maps are expected to carry Gaussian noise, we also evaluate the above minimum SNR for shot noise dominated experimental maps. All analysis remains the same, but instead of Gaussian noise, shot noise is added to the experimental maps (see “Methods”). Supplementary Fig. 2 shows the results of the same analyses, but with added shot noise, as in Fig. 6, for reconstruction in shot noise-based experimental maps. Similar trends are observed for shot noise analysis. However, the minimum SNR required for shot noise maps is found to be significantly higher as compared to Gaussian noise maps. We find 14.08 dB minimum SNR for 2D case (at shot noise factor = 0.2001) and 20.18 dB minimum SNR (at shot noise factor = 0.1001) for the 3D case. The shot noise maps contain noise proportional to signal magnitude, having more jitter at high magnetic field values. Therefore, shot noise affects the overall features in the NVMMs and hence, requires more SNR to be able to reconstruct accurately. Further, the residual noise, if any, in shot noise maps are highly correlated to, in terms of features, to the neuron NVMM that was subtracted from the experimental time series. Therefore, it does not lead to the complete removal of a neuron’s signature from the experimental time series, even when a spike for that neuron has been assigned. This effect further imposes high SNR requirements for shot noise map reconstruction.

## Discussion

In this work, we first show that APMF of the axon hillock in mammalian neurons can serve as a specific signature for detecting spiking activity in single neurons in a 3D volume of brain tissue. Previously reported imaging APMF of entire worm axon or reconstructing current-carrying wires^28,31,40,58^ suggests the usage of entire axonal APMFs, which would render 3D magnetometry-based imaging near impossible because of routes and long lengths of axons in brain tissue. We have estimated the magnitude of mammalian pyramidal neuron APMF for the axon-hillock segment to be 36 pT, which is two orders of magnitude larger than other locations of a neuron. Such specific APMF signatures thus allow reconstruction of single-neuron activity in a 3D tissue. Based on recent experiments and theoretical advancements, we believe that sensitivities of widefield magnetic diamond NVC imager will be within reach to image APMF signals. We have developed and presented an algorithm to find neuronal spike timing and location from 2D NVMMs. We show it is possible to perform spike activity reconstruction of hundreds to thousands of neurons located in a 2D layer or 3D volume. We also show the spatiotemporal limits of correct reconstruction to be in line with near-simultaneous firing of spikes at single-cell spatial resolution, provided sufficient sensitivity in the experiment.

We highlight the Gaussian noise resilience of the algorithm proposed. In cases of Gaussian noise, where the minimum SNR required is low in the range of −10 dB to −20 dB, an experimental setup with sensitivity nearly equal to peak magnitude of APMF will be sufficient to reconstruct neuronal spiking activity. Therefore, in widefield diamond NVC experiments, where spatial resolution, temporal resolution, and sensitivity per pixel are tightly coupled, application of the proposed algorithm on larger pixels might allow same reconstruction accuracy but an increase in temporal resolution or higher sensitivity experiments.

The approximation that the pyramidal neuron which has complex 3D current-carrying wire-like geometry can be simplified to a small localized current-carrying region like axon hillock, has certain limitations. Under certain neuronal arrangements, the higher intra-axonal current advantage of axon hillock segments maybe lost due to their relatively far off distance as compared to axonal segments from the NVC sensor plane (further discussed in Supplementary Note 3).

Apart from the requirement that the axon hillock be located close to the NVC sensor plane, there are two other limitations to be considered, and be further developed in future work. The proposed algorithm performance has been demonstrated with only important axon-hillock timepoints (see “Results”, population performance) in the dictionary and experimental maps are based on only important axon hillock timepoints. Implementation of the proposed algorithm for all timepoints would require improvements to handle large NVMM time series. The proposed algorithm works for ~0.5–1 spikes per second per neuron, in the range of sparse spiking in the cortices. However, under stimulus-evoked conditions, the cortical neurons can exhibit a large range of firing 10–90 spikes per second per neuron. Such high number of simultaneous spikes in experimental time series would pose a limitation and arises primarily due to inherently high correlations in the columns of the dictionary corresponding to nearby location neurons (within ~100 μms). We suggest that global residual minimizers like convex optimization of *l*_1_ norm can be used to improve the current algorithm.

However, in a shot noise dominated regime, the information in signal patterns is significantly lost due to the addition of noise correlated to signal magnitude. In this regime, signal-to-noise amplitude ratio should be nearly 10, to achieve a decent reconstruction. This SNR requirement would demand a sub-picotesla DC diamond NVC magnetometry, which has not been demonstrated yet. However, new techniques, with genetic manipulations for specific enhancement of the axon-hillock-associated APMF or converting the axon hillock associated current to an AC magnetic field, which can be detected with AC magnetometry where much lower levels of detection is possible^59–62^, would allow single-cell resolution mapping of APMFs with currently available magnetometry techniques.

The AP magnetic field signal is ~2 ms in timescale, which will fall in DC signal range as compared to diamond NVC measurement protocol, which can span ~10–2000 μs in time depending upon the coherence time *T*_2_ on the sensor^63,64^. As demonstrated in the former sections on the reconstruction of spiking activity, we suggest that DC vector magnetometry at, at least ~1 *pT μm* ^$-\frac{3}{2}$^ Hz^$-\frac{1}{2}$^ will be required to reach close to single-cell resolution spike detection, with our developed algorithm. In Barry et al.^28^, the authors demonstrated 34 *nTμm* ^$-\frac{3}{2}$^ Hz^$-\frac{1}{2}$^ DC-field sensitivity and they expect a 100-fold improvement with engineered diamond, Ramsey protocol, and optimized collection methods. Additionally, NVC metrology related work^58,60,65,66^ suggests the use of advanced quantum manipulation methods to reach closer to the quantum projection noise limited DC magnetometry. For example, Liu et al.^67^ demonstrated Ancilla assisted sensing to increase DC-field sensitivity and also performed a rejection of 1/f low-frequency noise in DC magnetic field measurements. Sub-picotesla magnetometry has already been demonstrated for AC field sensing^59^. Various expected DC-field magnetometry^64^-related research expect the ensemble diamond NVC sensitivity to reach volume normalized picotesla levels, where we expect to see at least, blurred single neuronal APMF signatures.

Considering other magnetometers for widefield APMF measurement, optically pumped magnetometers and SQUID magnetometers are two regularly used magnetometers that are much more sensitive than NVC magnetometers. However, both these techniques are usually single-point magnetometers and are difficult to extend to widefield microscale magnetic field imaging.

## Methods

### Simulations of axonal currents in the pyramidal neuron model

A realistic neuronal model^35^ was implemented in NEURON and used to simulate the membrane potential. Here, we briefly describe the simulation of membrane potentials. The cable equation that governs membrane potential is:

(3) $$\frac{1}{2\pi a\left(r_{i}+r_{o}\right)}\frac{\partial^{2}V_{\text{m}}\left(z,t\right)}{\partial z^{2}}=C_{\text{m}}\frac{\partial V_{\text{m}}\left(z,t\right)}{\partial t}+J_{\text{ion}}-J_{\text{m}}.$$

The discretized version of the equation was solved by implicit PDE solvers in NEURON. The temporal resolution of the voltage and current values available after the NEURON simulations was 10 μs. These data were imported and analyzed further in MATLAB (Mathworks) with custom written routines.

The pyramidal neuron used in the model has the following types of compartments: cell soma, axon hillock, action initial segment, unmyelinated region, and repeated regions of myelinated axon and node of Ranvier.

Intra-axonal current flowing across each segment of the neuron at a given time instant was calculated from the following equation:

(4) $$i_{\text{compartment },i}\left(t\right)=\frac{V_{\text{m}}\left(i,t\right)-V_{\text{m}}\left(i-1,t\right)}{r_{i}\frac{dl}{\pi a^{2}}},$$

where *i*, *i* – 1 denote the adjacent segments of the neuron. *r*_*i*_, *dl*, and *a* are resistivity, length, and radius of the segment, respectively.

### Simulations of APMF magnitude and experimental 2D NVMMs

By application of Biot–Savart’s law, we calculated the magnetic field $\vec{B}$ at a measurement point $\vec{r}$ by summing over different segments *n* of the pyramidal neuron.

(5) $$\vec{B}\left(\vec{r},t\right)=\sum_{j}^{N}ki_{j}\left(\vec{r_{j}},t\right)\frac{\vec{{\text{dl}}_{j}}\text{X}\left(\vec{r_{j}}-\vec{r}\right)}{{\left|\vec{r_{j}}-\vec{r}\right|}^{3}},\text{ where }\vec{B}={\left[B_{x}B_{y}B_{z}\right]}^{\text{T}},$$

where $\vec{dl_{j}}$ is a length vector along the segment, $\vec{r_{j}}$ is the position vector of the segment *j*, $\vec{r}$ is the measurement point, *i*_*j*_($\vec{r_{j}}$, *t*) is the current in the segment, *k* = *μ*_*o*_/4*π*, and the summation is over all segments *j* = 1,2, 3… *N* of the pyramidal neuron.

In order to estimate the magnitude of the mammalian APMF, a measurement point was selected perpendicularly below different segments of the pyramidal neuron at a standoff distance *d* from the centre of the compartment. The magnetic field at the measurement point was calculated by Eq. (5), above. APMF magnitudes at four different measurement points shown in Fig. 1e were selected as follows: perpendicularly below cell soma, below axon hillock, below longitudinal midpoint of the pyramidal neuron, and below the axon terminal end.

NVMMs are comprised of magnetic field values at multiple 2D spatial points calculated by varying the $\vec{r}$ vector (Eq. (5), above) at different points in the diamond NVC plane. NVMMs are 50 pixels × 100 pixels in size, with each pixel size equal to 20 μm × 20 μm. A time series of NVMMs are obtained by simulating the NVMM at different timepoints during AP propagation in the pyramidal neuron.

### Details of the proposed reconstruction algorithm

We solve for the inverse problem in Eq. (2), where *B* is the experimentally acquired 2D NVMM frames (with Gaussian noise or shot noise, see below in the section on Generation of spikes and time series of maps). For stating the linear inverse problem *AX* = *B*, we are treating *A*, *B* as scalar matrices. However, later while describing dot products of columns of *A* with any time instant of experimental map *B*, we refer to the notation **A**_*i*_ and **B**_*t*_ describing the vector nature of the magnetic field.

Mathematical symbols used to describe the algorithm are stated below:

1. *p*_x_ = 100 number of pixels of the NVC sensor in the *x* direction
2. *p*_y_ = 200 number of pixels of the NVC sensor in the *y* direction
3. *n* number of neurons in 2D plane or 3D volume
4. *n*_tp_ number of AP timepoints considered in the reconstruction (set to 3)
5. *A* dictionary matrix, size 3*n*_tp_ *p*_x_ *p*_y_ *Xn* (factor of 3 for **B**_x_, **B**_y_, and **B**_z_ components)
6. **A**_*i*_ columns of the dictionary of neuron *i* with the 3*n*_tp_ *p*_x_ *p*_y_ elements after concatenation of each component of *n*_tp_ timepoints. **A**_*i*_ denotes unit normalized vector form of **A**_*i*_
7. **B**_*t*_ concatenated experimental map at timepoint *t*,*t* – 1, *t* – 2… *t* – *n*_*tp*_ + 1, vector of length 3*n*_tp_ *p*_x_ *p*_y_
8. *T* is the threshold of projection value for detection of spiking. Threshold values of 10^–12^ and 10^–11^ were used for 3D and 2D cases, respectively.
9. *I*_B_ is a vector indicating the best-matched neuron index in every cycle of the algorithm, with values ranging from 1 to *n* or − 1 (no match). *I*_B_ is updated in different iterations of the algorithm with recursively changing experimental NVMMs.
10. *p*2 total number of successive spike-time event scan length for *I*_B_ indices, set to 3
11. *p*1 minimum number of occurrences of a neuron required in successive *p*2 elements of the *I*_B_ vector to be considered as a spike, set to 2
12. *p* running value of *p*1
13. Each column of **A**_*i*_ is set to |*ϕ*_*j*_〉, the NVMM of a particular neuron. This vector contains concatenated frames of multiple time instances (mainly corresponding to axon-hillock activity) and multiple directions (**B**_x_ **B**_y_ **B**_z_). Let *t*_1_ *t*_2_ *t*_3_… … … … … ..*t*_*n*_ be time instances of sampling. At each time *t*, we find resemblance of the experimental map to columns of the dictionary and assign the index of best-matched neuron to that time instant, accessed as *I*_B_(*t*). element of *I*_B_.

**Algorithm description as follows**

1. *p* = *p*2
2. While *p* ≥ *p*1
  2.1 While (new spikes are detected in the previous cycle of the loop or initialization)
    2.1.1 For loop (runs across timepoints for all experimental time instances or only specific time instances near last detected spike timepoint)
      Comment 1: At each experimental timepoint *t*, find which neuron *I*_B_(*t*) out of all neurons *n* resembles most to the experiment map **B**_*t*_
      Comment 2: Also, the value of the projection on normalized dictionary elements must be greater than a certain threshold (*T*) for the neuron to be selected or else we place –1 at *I*_*B*_(*t*)
        2.1.1.1 if max(**Â**_*i*_.**B**_*t*_) ≥ *T*, then $I_{\text{B}}\left(t\right)=k=argmax\;i\left({\hat{A}}_{i}.B_{t}\right)$ else if max(**Â**_*i*_.**B**_*t*_)<*T*, then *I*_B_(*t*) = –1
          end
          end loop
    2.1.2 Find consecutive occurrences of neuron *i* in the vector *I*_B_ for *p* times out of moving scan window of length *p*2
      Comment 3: If we find *p* occurrences of a matched neuron (*k*) in a continuous stretch of *p*2 elements in *I*_B_, a spike of the neuron *k* is detected.
      Comment 4: Precise timing of the *K* ^th^ neuron’s spike is determined by another search for time instant where subtracting *k* neuron’s signal leads to the maximum reduction in signal *B*. After subtracting **A**_*k*_ from the signal **B**_*t*_ for appropriate timepoints, **B**_*t*_ − **A**_*k*_, the residual is carried over as new signal *B* for the next iteration. Hence, we detect and subtract signatures of all spiking neurons one by one, at best timing location, until no further detection can be done, and the norm of a signal ∥**B**∥ at each time instant is less than threshold *T*. Notably, there are three parameters, namely T, *p*1 and *p*2 that control the output of the algorithm
    2.1.3 On detection of a spike of neuron *k* find argument *t*_0_ that maximizes
      (Â_*k*_.**B**_*t*_0__, *where t*_0_ *ranges from t – n*_*tp*_ *tot* + *n*_*tp*_ – 1 & only where *I*_B_(*t*_0_) equalsi:). Here, *t* corresponds to the timepoint where regular occurrences of a particular neuron index were found in the last step.
    2.1.4 Choose the argument of this maximum in 2.1.3 as spike timing *t*_spike_ of neuron *k* followed by subtraction of signature of this spike from the experimental map as described in comment 4. Set **B**_*t*_ = **B**_*t*_ − *A*_*k*_ and record spike of neuron *k* at *t*_spike_ time instant. Comment 5: After subtracting NVMM corresponding to a particular spike of a neuron, when we go to recalculation of *I*_B_ from the new experimental map, we do not evaluate *I*_B_ indices over all timepoints of the experiment. We revaluate *I*_B_ only within the timepoints which have changed due to the subtraction of NVMM of the last spiking neuron.
      End of 2.1 While loop.
3. Decrement p = p–1
  End of 2. While loop

### Running algorithm and setup of dictionary

The algorithm was quantified in two different cases—2D case, where the neurons are located in a plane parallel to the diamond NVC layer, and a 3D case, where the neurons were distributed in 3D volume, randomly oriented, mounted over a diamond NVC layer. In the 2D case, the dictionary matrix is comprised of individual NVMMs of 80 neurons laterally shifted by 20 microns. In the 3D case, there are total 6250 different NVMMs from randomly oriented neurons in the 3D volume of 1mm × 2 mm× 70 μm. The placement of cell somas/axon hillocks was done in a grid-like manner by placing 25 × 25 neurons in each plane parallel to diamond NVC layer, and stacks of ten such planes with varying perpendicular distance, z coordinate, from the diamond NVC layer. The spatial resolution of this grid was 40 μm × 40 μm × 7 μm in *X*, *Y*, and *Z*, respectively. After the placement of cell soma, the direction of the neuron was randomly chosen from ten different orientation angles between 0 to 90°. The corresponding NVMMs were added to the 3D case dictionary.

The experimental map was constructed as a convolution of the spike timing vector and individual NVMMs time series. The timing resolution was kept at 0.5 ms, and the total simulation time was set to 600 ms. Spike timing was assigned by the method specified in the next section.

### Generation of neuronal spikes and time series of maps

The probability of spike of a neuron at a time instance is given by *f*, a factor that controls the spatial and temporal density of firing. Higher *f* will lead to more spatially and temporally sparse firing. For each neuron, *f* is a binomial probability. At each time instant, we generate a uniform random number *r*_*i*_ ranging between 0 and 1 for each neuron *i*. A spike occurs in neuron *i*, if *r*_*i*_ > *f*. Thus, the spike times of each neuron are independent of each other. After assigning spikes by the above-stated method, for each neuron, to simulate a refractory period^44,46^, a neuron is not allowed to spike for a period of 5ms following a spike. The factor *f* for the 2D case was adjusted to be 0.994 and for the 3D case to be 0.9996 so that sparse firing in the population, as in the cortex is observed^68^.

For 3D performance, some additional spike times, other than multiple spikes within the refractory period, were removed before applying the algorithm. Spikes of neurons of following two types in the 6250 element 3D Dictionary (see the section “Running algorithm”) were removed, and hence these neurons produce no spikes.

Type 1—A neuron whose RMS value of 1D vector (concatenated 2D NVMM) element in the dictionary is less than the threshold (1 pT for 3D case). If we assign spikes to these neurons, they are always rejected in the threshold step of the first iteration itself while running the proposed algorithm. Hence, spikes of these neurons are pre-removed before running the algorithm.

Type 2—2D NVMMs were generated by summing neuronal intra-axonal currents in Bio–Savart expression for each *z* plane and a random orientation (see the section “Running algorithm”). However, for each *z* plane and orientation angle, the *XY* grid was simulated by translation of the map along *x* and *y* axis. In this translation, some neurons can be shifted to the extent that the axon-hillock-related signatures do not fall directly above the diamond NVC layer. Hence, these neurons lack important axon hillock patterns in their NVMMs and are significantly less in their RMS values (in agreement with high axon-hillock contribution). Spikes of these neurons are removed, as they are never detected by the algorithm. In this dataset of 3D dictionary generation, these neurons are ~40% in number.

Type 1 and Type 2 have a large number of common neurons, whose axon-hillock segments are displaced off the NVC layer. Only their long axonal parts fall perpendicularly over the diamond NVC layer.

However, their individual 2DNVMs of both types are always present in the dictionary during the run of the proposed algorithm.

### SNR calculations

We add Gaussian or shot noise to the experimental time series of NVMMs in the following manner:

**S** = [**B**_*t*_ **B**_*t*+1_ **B**_*t*+2_ …] is the concatenated 1D vector of all 1D **B**_*t*_ experimental maps at different timepoints

**S** ^noise^ is the 1D vector with noise added to each element of vector **S** *η* is Gaussian or shot noise factor

*randn* MATLAB function was used to generate a Gaussian random variable with zero mean and standard deviation 1

*rms*(**S**) root-mean square of all elements of vector **S**

For Gaussian noise, each element of S^noise^ is given by

(6) $${\text{S}}_{i}^{\text{noise}}={\text{S}}_{i}+\eta^{*}rms{\left(\text{S}\right)}^{*}randn\text{.}$$

For shot noise, each element of **S** ^noise^ is given by

(7) $$S_{i}^{\text{noise}}=S_{i}+\eta^{*}{\left|S_{i}\right|}^{*}randn.$$

SNR is given by

(8) $$SNR=20^{*}\log_{10}\frac{rms(\text{S})}{rms\left({\text{S}}^{\text{noise}}-\text{S}\right)}.$$

To be noted, shot noise maps are dependent on per pixel magnitude, |**S**_*i*_| and hence, perturb the experimental maps more at pixels where magnetic field is high. However, Gaussian noise maps get the same standard deviation noise added depending on the term *rms*(**S**), which remains constant for different elements ${\text{S}}_{i}^{\text{noise}}$ of vector **S** ^noise^. Also, noise factor values for lateral and axial cases in experimental maps can not be directly compared, but their SNR values can be compared.

## Supplementary Material

Supplementary information is available for this paper at https://doi.org/10.1038/s42005-020-00439-6.

Supplementary Material

## Data Availability

The datasets generated during and/or analyzed during this study are available from the corresponding author on reasonable request. The pyramidal neuron computational model^35^ used in this study is available at ModelDB Accession code: 123897.

## Code availability

MATLAB codes for data processing, analysis, and algorithm implementation performed in this study are available from the corresponding author on reasonable request.
