# Supplementary material for: Axon hillock currents enable single-neuron-resolved 3D reconstruction using diamond nitrogen-vacancy magnetometry
Source: Commun Phys. Author manuscript; Available in PMC 2020 Oct 16. (PMC7116192; doi:10.1038/s42005-020-00439-6)
Supplement: Supplementary Material [file EMS96524-supplement-Supplementary_Material.pdf]

SUPPLEMENTARY INFORMATION FILE

Title:

Axon hillock currents enable single-neuron-resolved 3D reconstruction using diamond nitrogen-vacancy magnetometry

Authors: Madhur Parashar[1], Kasturi Saha[2]*, Sharba Bandyopadhyay[3]*

*corresponding authors

2*kasturis@ee.iitb.ac.in

3*sharba@ece.iitkgp.ac.in

[1]School of Medical Science and Technology, Indian Institute of Technology Kharagpur, Kharagpur 721302, India

[2]Department of Electrical Engineering, Indian Institute of Technology Bombay, Powai, Mumbai 400076, India

[3]Department of Electronics and Electrical Communication Engineering and Advanced Technology Development Centre, Indian Institute of Technology Kharagpur, Kharagpur 721302, India

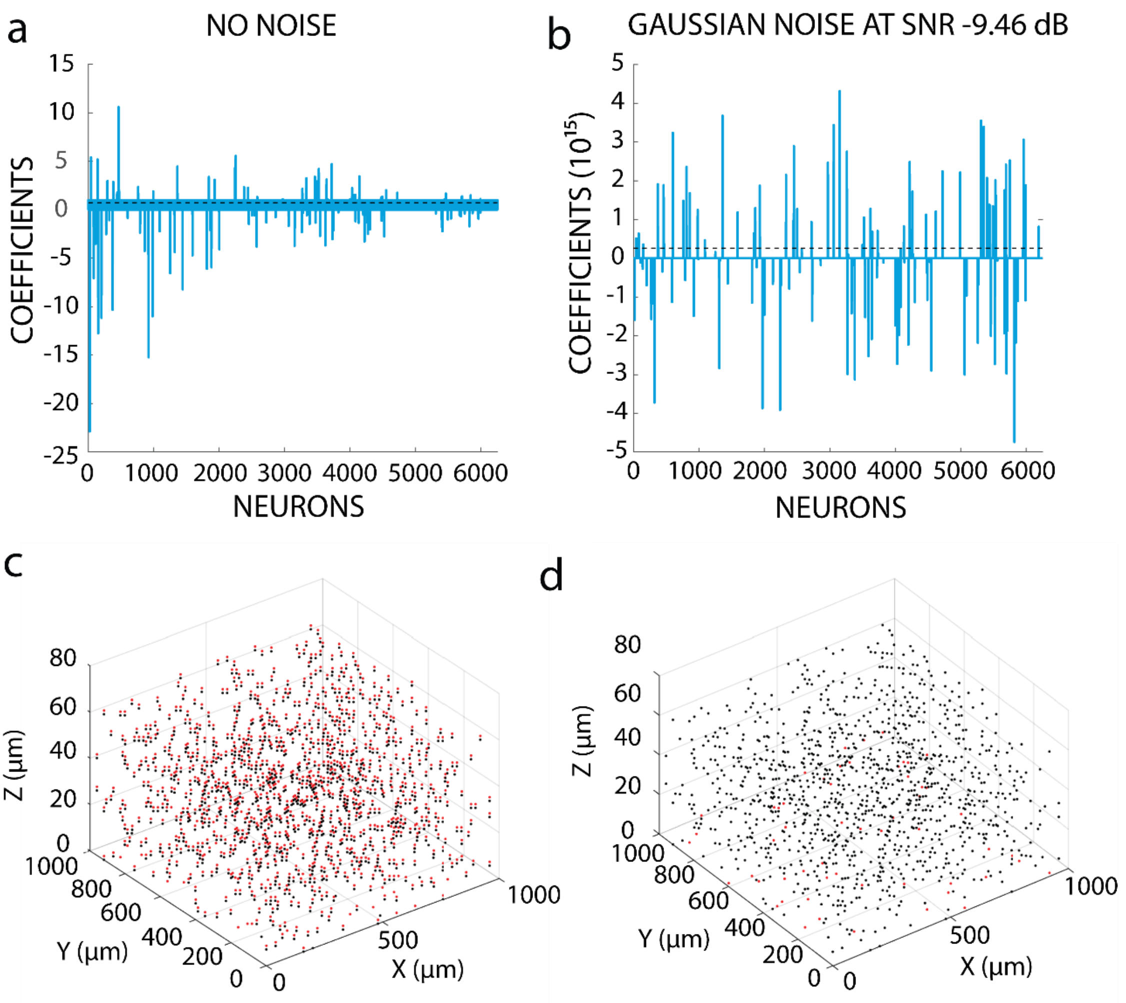

Supplementary Figure1: A pseudoinverse-based reconstruction of simulated 3D cortical activity.

(a)-(b) Coefficients after pseudoinverse calculations in no noise case (a) and with -9dB gaussian noise (b) added to experimental 2D diamond nitrogen-vacancy magnetometric maps (NVMMs). The horizontal line shows threshold level for considering an action potential (AP) instance. **(c)-(d)** 3-D reconstruction, in no noise case (c) and with gaussian noise added case (d). Black dots represent actual AP instances and red dots represent AP instances obtained from pseudoinverse reconstruction. The reconstruction is nearly 100% percent accurate when no gaussian noise is added but has very low correct classification percentage when gaussian noise is added.

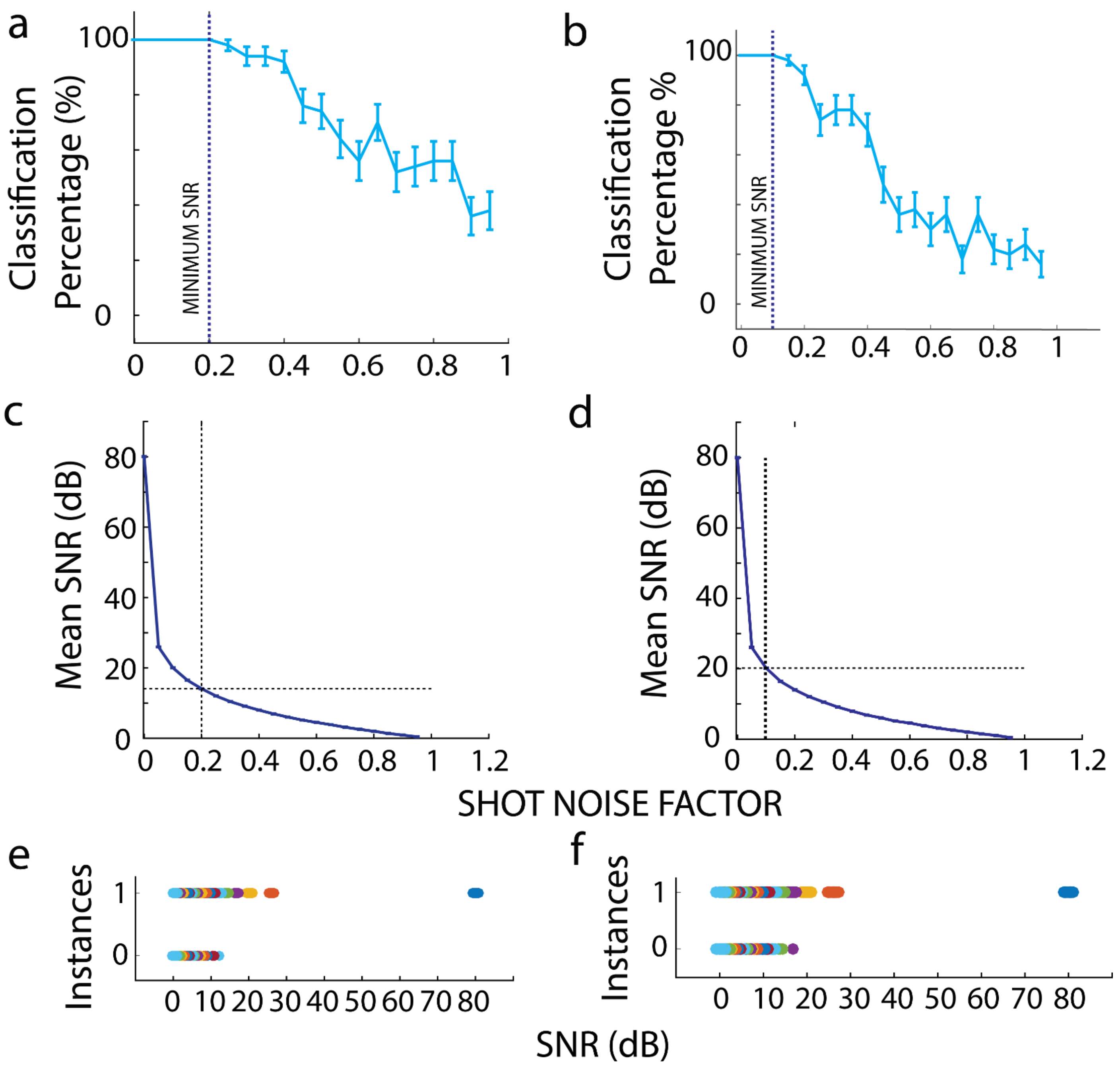

Supplementary Figure 2: Resolvability of spatially and temporally close action potentials at varying shot noise levels.

Minimum required signal to noise ratio (Min. SNR) is shown, for resolving action potentials (APs) from laterally and axially separated nearby neurons from 2D diamond nitrogen-vacancy center magnetometric maps (NVMMs) added with shot noise (a) Plot of correct classification percentage versus shot noise factor, a factor to control levels of shot noise added to NVMMs, for lateral separation case (two neurons ,$10\mu m$ apart, spike time difference $0.5ms$). Min. SNR has been marked as the point where the standard deviation (standard error bars shown) of correct classification percentage drops to zero (in 50 repetitions) (b) Plot of current classification percentage versus shot noise factor for

axial separation case (two neurons, $7\mu m$ apart, spike time difference $0.5ms$). Vertical line marks point of Min. SNR. (c) Plot of mean signal-to-noise (SNR) ratio(dB) versus shot noise factor for lateral separation case. (d) Plot of mean SNR (dB) versus shot noise factor for axial separation case. Parts a,b,c and d plots share the same X axis title– shot noise factor **(e)-(f)** Example individual instances of correct(1)/ incorrect(0) reconstruction by proposed algorithm versus SNR(dB) for lateral separation case (e) and for axial separation case (f). A clear shift in certainty of reconstruction is observed at SNR above Min. SNR. Also, colors where randomly assigned for dots corresponding to different SNR and individual dots might be further separated on SNR axis at finer scale, as different trials with same shot noise factor generate slightly varied SNR. All errorbars in this figure represent one standard error over 50 independent trials. For each value of shot noise factor, 50 independent NVMMs were produced and correct/incorrect classification was performed for each of these NVMMs by the proposed algorithm.

Simulation Type	Actual spike instances (a)	Algo marked spike instances (b)	Correctly Marked by Algorithm (c=intersect (a, b))	Percentage performance (c/b) x100	Percentage performance (divided into 50ms bins) Mean+/-std error	SNR (dB)	f
2D without noise	579	456	382	83.77	83.61 +/- 2.17	--	0.994
2D with noise	579	474	396	83.54	83.82+/- 2.09	-11.87dB	0.994
3D without noise	1727	898	612	68.15	67.77+/-1.41	--	0.9996
3D with noise	1727	727	523	71.94	71.73+/-1.19	-9.46 dB	0.9996

Supplementary Table 1: Population performance data in case of dictionary elements based on time points 3.5ms, 4ms, 4.5ms.

SNR denotes signal-to-noise ratio of experimental 2D diamond nitrogen-vacancy magnetometric maps (NVMMs). Variable a and b refer to actual number of spike or action potential instances and number of spike instances marked by the algorithm respectively. Variable c refers to number of correctly marked spike instances by the algorithm evaluated by taking the intersection of sets of spike instances corresponding to actual spikes (a) and algorithm marked spikes (b). Variable f refers to the factor for controlling the spatial and temporal density of spikes in experimental NVMMs simulations. Percentage performance of the algorithm is quantified as (c/b) times 100.

Simulation Type	Actual spike instances (a)	Algo marked spike instances (b)	Correctly Marked by Algorithm (c=intersect (a, b))	Percentage performance (c/b)x100	Percentage performance (divided into 50ms bins) Mean+/-std error	Signal-to-noise ratio SNR (dB)	f
2D without noise	579	499	425	85.17	85.45+/-1.37	--	0.994
2D with noise	579	686	389	56.71	57.12+/-2.04	-11.8375	0.994
3D without noise	1727	63	33	52.38	40.39+/-9.84	--	0.9996
3D with noise	1727	903	2	0.22	0.23+/- 0.22	-9.4641	0.9996

Supplementary Table 2: Population performance data in case of dictionary elements based on time points 3ms, 3.5ms and 4ms

SNR denotes signal-to-noise ratio of experimental 2D diamond nitrogen-vacancy magnetometric maps (NVMMs). Variable a and b refer to actual number of spike or action potential instances and number of spike instances marked by the algorithm respectively. Variable c refers to number of correctly marked spike instances by the algorithm evaluated by taking the intersection of sets of spike instances corresponding to actual spikes (a) and algorithm marked spikes (b). Variable f refers to the factor for controlling the spatial and temporal density of spikes in experimental NVMMs simulations. Percentage performance of the algorithm is quantified as (c/b) times 100.

Simulation Type	Actual spike instances (a)	Algo marked spike instances (b)	Correctly Marked by Algorithm (c=intersect (a, b))	Percentage performance (c/b)x100	Percentage performance (divided into 50ms bins) Mean+/-std error	SNR (dB)	f
2D without noise	579	456	382	83.77	83.61 +/- 2.17	--	0.994
2D with noise	579	474	396	83.54	83.82+/- 2.09	-11.87dB	0.994
3D without noise	1727	915	636	69.51	70.18+/-1.39	--	0.9996
3D with noise	1727	743	534	71.87	71.86+/-1.03	-9.46 dB	0.9996

Supplementary Table 3 – Population performance data with Pearson correlation coefficient criteria.

The experimental dataset and dictionary used for the simulations were same as that of Supplementary table 1 and Figure 4. SNR denotes signal-to-noise ratio of experimental 2D diamond nitrogen-vacancy magnetometric maps

(NVMMs). Variable a and b refer to actual number of spike or action potential instances and number of spike instances marked by the algorithm respectively. Variable c refers to number of correctly marked spike instances by the algorithm evaluated by taking the intersection of sets of spike instances corresponding to actual spikes (a) and algorithm marked spikes (b). Variable f refers to the factor for controlling the spatial and temporal density of spikes in experimental NVMMs simulations. Percentage performance of the algorithm is quantified as (c/b) times 100.

Supplementary Note 1

Discontinuities in intra-axonal current profiles in Figure 1d and Figure 1e

The voltage propagation data looks smooth and continuous (Figure 1a), however, there are certain nearby compartments that are always isopotential. We find that these adjacent isopotential compartments are always aligned to places where segment type changes and also, in relatively long same type segments. It is important to mention here that the partial differential equation solver (PDE) solver, NEURON is routinely used to simulate neuronal cable theory equations. The intra-axonal current density maps are proportional to the derivative along the segments axis (first order partial spatial derivative) of the voltage propagation maps, and hence, they show a sudden dip to zero and then immediate jump to larger value, amidst continuous set of values. These peaks have not been removed, as we found these were inherent to NEURON model and further, as the magnetic field maps are spatial integrals (across all segments) of the current map, these peaks are quickly averaged. We don’t negate the effect of these spikes in current profile, but we find the magnetic field profiles fairly smooth.

We believe that these iso-potential segments are included in NEURON to create a boundary value condition at segments where the segment type, radii, and other physiological properties of segments change, for the sake of numerical stability.

Supplementary Note 2

Decreased performance when including timepoint 3ms maps in Figure 2

Here, we explain the details of not including NVMM at timepoint 3ms (Figure 2 b,h,n) in reconstruction dictionary. Including NVMMs at timepoint 3ms, along with timepoint 3.5ms and 4ms yields poor performance in reconstruction (Supplementary Table 2). We found that the auto-correlation of an individual NVMM at a given timepoint, say 3.5ms,

to its nearby timepoints is important to reconstruction. By including NVMM with two major lobes going sign inversion (Figure 2), the auto-correlation map looks like a peak at zero lag and surrounded by negative dips at on both sides. But when, we include NVMMs where all major lobes have the same sign, these auto-correlations maps would have positive components only. However future methods should exploit the strong component in NVMM at timepoint 3ms.

Supplementary note 3

Limitation to approximating complex 3D current carrying wire geometry to localized tapered cylindrical geometry

In our 3D volume reconstruction, we have addressed a specific class, but not completely general case, of 3D neuronal arrangements. While assigning neuronal location and orientation, we assign somas in a grid like fashion in $1mmX2mmX70\mu m$ volume, with the grid resolution as $40\mu mX40\mu mX7\mu m$. Now, keeping the coordinate origin at each soma, we have assigned a random rotation ($10°$ to $90°$, in steps of $10°$) to each of the neurons, soma and axon. The transformation leads to a rotated configuration of the neuron, with the cell soma at the same assigned place. Further, we build a dictionary from axon hillock signatures of differently oriented neurons and quantify performance.

However, the lateral dimension of this pyramidal neuron is quite large ~$2000\mu ms$. Cases, for example, pyramidal neuron perpendicular to the NVC sensor plane with axon hillock nearly full length (~$2000\mu ms$) far off from the sensor plane and axonal segments close to the sensor plane, can't be reconstructed with current proposed algorithm.

We have dealt with a specific class where soma, and hence adjacent axon hillock segments, are close to the sensor plane (~$70\mu ms$). Hence, the higher intra-axonal current advantage of axon hillock segments is not lost due to their relatively far off distance as compared to axonal segments from the NVC sensor plane. We have added this discussion in form of a limitation to the reconstruction. Further, as long as axon hillock segment are sufficiently close to the sensor plane, we show that changes in their activity profile, with different orientations, can still be reconstructed using the current algorithm.

Additionally, we find that the Axon hillock signature involves a unique sign flip of major lobes, as seen in NVMMs from changing from timepoint 3ms to 3.5ms Figure 2 . While our algorithm does not leverage this feature, it can always be used to distinguish Axon hillock signature from the rest of axonal regions' signature, irrespective of

orientation of the neuron, provided sufficient SNR. Therefore, we can still approximate complex geometries to small localized region, around axon hillock, in this reconstruction problem using other algorithms that successfully match the sign inversion of magnetic field in time (NVMMs timepoint 3ms to 3.5ms Figure 2).
